# Creating health-promoting spaces for change within the economy: the role of food co-operatives in England

**DOI:** 10.1093/heapro/daag038

**Published:** 2026-03-19

**Authors:** Amy Barnes, Maddy Power, Kelli Kennedy

**Affiliations:** Public Health and Society, Health Sciences, University of York, York, YO10 5NB, United Kingdom; Department of Public Health, Environment and Society, London School of Hygiene and Tropical Medicine, London, WC1H 9SH, United Kingdom; Public Health and Society, Health Sciences, University of York, York, YO10 5NB, United Kingdom

**Keywords:** co-operatives, co-ops, power, control, health promotion, economy, inequality, economic change, democratic decision-making

## Abstract

Public health is in crisis, with health inequalities and environmental degradation reflecting failures and uneven power in our economy. There is interest in the potential of co-ops to address these issues. However, limited research explores if or how co-ops could reshape power dynamics towards health promotion within our current economy or wider transformation of it. Drawing on an understanding of health as the ‘capabilities for everyday living’ and power to live a life you value, as well as the concept of ‘space’, this article explores power dynamics in and around co-ops and how these shape co-ops’ role in promoting health. While focusing on food co-ops and the economy in England, the findings offer broader learning. Based on a mixed qualitative methods study (March–July 2024), we show how three types of co-ops (established-scaled worker co-ops, ‘emanant’ worker co-ops, and community organizing co-ops) *all* act as enabling spaces for distributing power within the current economy; specifically, creating conditions for members to exert control in their lives (at work, in food systems and local areas), including through meaningful participation, access to affordable food and a more equal income. Co-ops also create other opportunities for meaningful connection and purpose which co-op members value as important for their wellbeing. However, the capacity of co-ops to exert wider transformation is constrained by their wider political, economic and social context, including the relative isolation in which they operate. Without broader collective mobilization to scale out, their full public health potential is unlikely to be realized.

Contribution to health promotion

- First paper to explore the role of co-op power dynamics in promoting public health.
- Shows how three different types of co-ops can *all* create conditions for distributing power so that members can exert control in their lives (at work, in food systems, and local areas), including through meaningful participation (i.e. with status and recognition), access to affordable food and a more equal income.
- Co-ops can also create other opportunities for meaningful connection and purpose, which co-op members value as important for their wellbeing.
- For co-ops to reach their full health-promoting potential, they would need greater reach and influence across society.

## Background

Public health is in crisis globally, with high costs of living and poverty, stark health inequalities and environmental injustice reflecting failures and uneven power in our economy: we are not producing and providing for one another effectively (WeALL 2025). Part of the issue is arguably the predominantly extractive way in which our economic institutions are governed, which tend to concentrate, rather than broadly distribute, wealth, income, power, and status (Wilkinson and Pickett 2009). This contributes to the situation we see in many high-income countries in which living costs are unaffordable for a significant number of people (e.g. through low pay, insecure work, and high costs of housing, food and other essential goods) (Hickson 2024), undermining people’s ability to exert agency and control over decisions and actions that impact their lives and thus their ability to be healthy. Economic transformations are urgently needed at global and national levels to address the public health and inequality crisis.

At a national level, redistributing economic gains (e.g. through taxation and social security benefits) (Guinan and O’Neill 2020), mitigating environmental damage, and treating the health harms of livelihood insecurity (e.g. via mental health services, health care, advocacy support) are common ways of trying to promote health in the economies of high-income countries. Redistributive approaches are recognized as important and ‘life-changing for those who benefit from them’, but have, arguably, failed to deliver substantive change for public health and can be subject to political whims (Raworth 2017). In England, for example, there have been successive, ideologically-driven changes in social security policy, which have created precarity for families and undermined mental health (Power *et al*. 2023). In consequence, complementary action is needed to ‘design out’ inequality-generating institutions in the economy: growing those that more fairly predistribute income, social status, agency, and control (Young 2002), and which thus enable, rather than constrain, people’s health; which we define here, as in Ottawa Charter of Health Promotion, as people’s ‘capabilities for everyday living’, including people’s ability to realise aspirations, satisfy needs, and cope with their environment: it is the power to live a life you value (WHO 1986).

### Co-ops as spaces for health-promoting change?

There has been renewed interest in the role of co-ops as ‘alternative’ institutions that can meet these needs, by fostering greater equality and democratic participation within national and global economies (Raworth 2017, Spicer and Kay 2022). International interest is reflected in the United Nations General Assembly declaration that 2025 was the second ‘International Year of Co-operatives’ (IYC2025) (UN 2025).

Co-ops are people-centred enterprises, which are jointly-owned and democratically controlled by their members, who come together to realise common economic, social, and cultural needs within a value framework of fairness and social justice (ICA 2024). There are multiple types of co-ops. They can take different forms depending on who the members are and which sector of the economy they operate in (e.g. food, housing, social care, media/journalism, finance), but their democratic, member-driven nature is a clear differentiating factor (UN 2024). Some co-ops are owned and run by the people who work there, while others are owned and run by people who belong to a particular geography or community of interest, with many hybrid forms.

There is a rich scholarship on co-ops, particularly in the field of organizational studies, and on worker co-ops specifically. Existing work draws attention to the economic benefits of worker co-ops (e.g. their higher ‘survival rates’, worker productivity, and satisfaction) and sometimes imperfect ways in which they can promote democratic participation within the economy (Sobering 2016, Sutton 2019, Powell 2021). This includes challenges of maintaining meaningful participation over time due to ‘mission drift’ and as co-ops grow in size (Cornforth 1995, Powell 2021), as well as questions about their potential for economic transformation (Spicer and Kay 2022). Existing literature also highlights how co-ops often emerge from social networks or movements at particular ‘moments’ in history, often during economic crises (Spicer and Kay 2022). For economic transformation, cross-coordination and the development of ‘co-operative ecosystems’ act are thought to be important mechanisms to help co-ops thrive and ‘reach a scale that has impact’ (Allen *et al*. 2003, Hoover and Abell 2016:2; Sutton 2019).

Less research has focused on the role of co-ops in relation to the public health and inequality crisis discussed earlier in this article (Spicer and Kay 2022). While Wilkinson and Pickett (2009) highlight a potential health-promoting role for co-ops in their seminal ‘Spirit Level’ work on the negative health effects of income inequality, there has been little further work on this topic within the field to connect public health understandings to issues of power, or to insights on co-ops within organizational studies. We thus know relatively little about the extent to which co-ops *pre*distribute and reshape power dynamics towards health promotion within our current economy or how they might be able to bring wider transformation of it.

### Co-ops, power and ‘space’

As suggested by the discussion above, any analysis of the potential of co-ops to mitigate inequality and promote health must directly consider power dynamics within and surrounding them, and how these might enable or constrain people’s capabilities for health. For this, we suggest that the concept of ‘space’ is a useful analytical tool to map the power geometry of co-ops. Spatial metaphors are common in literature on co-ops. They have been variously described, for example, as ‘alternative spaces’ (Spicer and Kay 2022), ‘capability spaces’ (Nunes 2015) and ‘counter spaces’ (Nicolosi 2020) with emancipatory potential. Yet, only a few studies apply the concept of space in-depth to co-operatives (Powell 2021). We suggest that there is value in drawing on the idea of ‘space’ as it has been used in diverse literatures on power (Gaventa 2006, Massey 2009), the economy (Harvey 2002), and democratic participation (Cornwall 2002), and to combine it with existing literature on co-operatives, because ‘space’ is a useful, unifying concept for analysing power within wider social, economic, and political processes (Massey 2009, Powell *et al*. 2021). Massey, in particular, developed space as a valuable concept for analysing processes of power (see e.g. Massey 2009), and the concept has started to be used to explore the configurations of power (re)produced in initiatives to promote health (Renedo and Marston 2015, Powell *et al*. 2021).

In terms of the value of the concept in understanding power, existing literature highlights that, while ‘organizations’ may typically be seen as bounded and fixed, ‘spaces’ are open and porous, with a constantly changing assemblage of relationships, which reflect power dynamics over time (Cornwall 2002, Massey 2009): or what Lefebvre (1991:110) calls traces of the ‘generative past’. Applying the concept of space to co-operatives means conceptualizing them as relational and political products that are always ‘under construction’ and ‘being made’ (Massey 2009:17). This multidimensional understanding of space—as political, relational, and temporal—provides a valuable heuristic for examining configurations of power, and thus if and how co-operatives create conditions for people to develop capabilities to be healthy within the economy, or (re)produce an inequitable and unsustainable status quo.

In the political dimension, cooperative spaces can be understood as a constant ‘interplay’ of contestation, collaboration, and difference and which cannot be entirely cleared of wider assumptions, meanings, and power dynamics that exist within society and the global capitalist economy (Cornwall 2002, Powell *et al.* 2021). This draws attention to the importance of understanding how different cooperatives manage these tensions; how internal dynamics may exclude, transform, or entrench pre-existing racialized, gendered, or classed inequalities and occupational segregation (Sobering *et al*. 2014, Sobering 2016); and the way in which the wider capitalist sociopolitical environment, as well as a cooperative’s ‘founding moment’, may affect how inequality is shaped within it over time (Sobering 2016 , Spicer and Kay 2022). While some cooperatives may position themselves as sites of ‘radical possibility’ at the margins, which members make and shape for themselves (Radical Routes 2025) existing literature reminds us that these spaces will often be residual to the state and ‘framed by’ the state’s existence (Cornwall 2002). Indeed, most cooperatives must work within, what Plender calls, the ‘contradiction’ of ‘being beyond and simultaneously within’ the current order (Homs and Narotzky 2019:133 in Plender 2022).

In the relational dimension, the *impetus* for developing co-operatives—how and by whom they are produced (i.e. their origins and continuing motivations of members)—is a significant consideration, shaping the power dynamics operating within them: determining who can enter, on what terms, and with what identity, interest or authority; what is say-able or do-able; and the relationships made (Cornwall 2002, Gaventa 2006, Sobering *et al*. 2014, Sobering 2016; Powell 2021). Impetus can also shape the extent to which existing forms of power (the ‘old rules of the game’) shape participation: silencing or preventing people from entering (Sobering 2016, Powell 2021; Powell *et al*. 2021). Yet, co-operatives can be ‘claimed’ as ‘spaces of possibility’ if groups previously excluded lever access based on their own terms of participation (Cornwall 2002, Sobering 2016, Powell 2021; Powell *et al*. 2021).

Finally, *durability* is a key temporal element, referring to the quality of permanence and relational strength that holds and keeps co-op members together over time, with durability also affected by access to material resources and relations with the state and civil society over time (Powell 2021). This includes, for example, consideration of co-operatives’ reliance on the state for funding or legal status within the economy and how their durability might be affected by policy changes, such as the ‘contracting out’ of services or during periods of austerity (Huckfield 2022).

In this paper, we apply these three characteristics of space—focusing on the wider power environment, impetus, and durability—to understand the configurations of power operating in and around co-ops and explore how these enable or constrain the development of people’s capabilities to be healthy or, conversely, reproduce the status quo. Drawing on qualitative data from our study focused particularly on co-ops relating to food in England, we highlight how three different types of co-ops all create a power geometry that contributes to health promotion through *similar* means: creating conditions for members to exert control over their material and political environment, as well as creating other opportunities for meaningful connection and purpose which co-op members value as important for their wellbeing. Wider transformation is, however, constrained by the current context in which cooperatives operate.

## Case study context: co-ops related to food in England

Given the breadth of cooperative activity, we anchor our research in a particular aspect of the economy: food. We chose to focus on co-ops related to food given that issues of poverty, inequality, and environmental decline (as highlighted above) are all particularly evident in relation to how food is governed and experienced. Not only are extractive and commercialized systems of production and distribution unable to equitably meet people’s most basic nutritional needs, as evidenced by rates of under- and over-nutrition across the globe, and high levels of diet-related non-communicable diseases (Maguire and Monsivais 2015, Sisnowski *et al*. 2017, Barnhill *et al*. 2018), but they also contribute to unhealthy conditions for food workers (Parsons 2020), to water, soil, air pollution, and climate change (Willett *et al*. 2019, FAO and UNICEF 2022) and erode democratic participation and control in the production and consumption of food (Levkoe 2011). Food is intrinsically tied to human wellbeing with strong cultural value and maintaining control and participation in relation to it is paramount if we are to protect and promote the health needs of people and the planet.

We also chose to focus on the economy—how people produce and provide for one another—in England, not only because this is where we, as authors, are all situated, but also because England has a long history of cooperative activity grounded in collective mobilizations around food. The cooperative movement is widely regarded to have emerged in Lancashire, England, when The Rochdale Society of Equitable Pioneers was founded in 1844 by a group of working-class men who sought to collectively act against issues of food adulteration and high prices (Fairbairn 1994, ICA 2024). The ‘Rochdale model’ involved the pioneer members pooling their resources to buy wholesale, to provide access to basic food goods at lower prices, and with profits and decisions shared, as every customer became a cooperative member (Fairbairn 1994, ICA 2024). Forms of cooperative activity have continued in England ever since, albeit with periods of growth and decline, and with a relative dominance of retail food cooperatives, owned and controlled (at least in part) by customer members (Deblangy 2023, Webster 2023).

Discontent with politics and the state of the economy in the 1970s saw a period of cooperative expansion, as political activists looked for solutions through the creation of worker co-ops, including in wholefoods, printers, and radical bookshops (SEA 2018). This was supported politically at the time by national cooperative development programmes of the Labour government. However, as neoliberal ideas came to dominate politics and the economy in England, supportive infrastructure for cooperatives has been dismantled, starting under Thatcher and continuing with New Labour, who promoted more hierarchical and individually-, rather than democratically-controlled ‘social enterprises’ in place of co-operatives and as a means of low-cost public service delivery (Powell 2021; Huckfield 2022).

At the time of writing, there was a relative ‘absence of legislation and policy, institutional support, advice, incentive and promotion’ of different types of co-ops by the state in England (Lawrence *et al.* 2018), though the 2024 Labour government pledged to double the size of the co-operative sector (Labour Party 2024). There has also been renewed civil society mobilization around co-operatives, as reflected in the 2022 establishment of a new UK federation of worker-led cooperatives, many of which are involved in the food system, and who collectively commit to build a more equitable and sustainable world (workers.coop 2025).

## Methods

This study, which was carried out between March and July 2024, employed an exploratory qualitative research design, underpinned by an interpretive/constructivist methodological tradition (Denzin *et al*. 2023). Following Allen *et al*. (2003), this approach allowed for in-depth, contextual understanding of how co-op members constructed, experienced, and negotiated power dynamics in their co-ops and in relation to the wider economy, which was critical for assessing health promotion potential. The design used mixed qualitative methods (interviews, site visits, and document analysis) across different cooperatives in England to capture a broad range of experiences.

The study adopted a purposive sampling strategy to recruit participants who worked in or in relation to different co-ops relating to food. At the time of starting the study, we knew that there was diversity in the co-op sector (e.g. geographical location, size, number of members, relationship to food). We drew up a sampling frame to purposefully identify potential participants from a diversity of co-ops, based on these initially identified dimensions of difference. The dataset included semistructured face-to-face or online interviews with 12 participants who either worked in (*n* = 9) or with food cooperatives (*n* = 3), including 8 male and five female participants. All participants had been involved in their organization or co-op for more than a year, though many of the participants we spoke to had been involved with food co-ops for a much longer period (e.g. 5–10 years).

Interviews explored topics such as what co-ops do and how they operate day-to-day, how the co-ops that they had experience of engaged with co-operative principles, perspectives on how co-ops shaped different dimensions of public health and wellbeing and why, factors that supported or undermined what co-ops do, as well as following up any topics and issues identified as significant to health or relating to issues of power within each interview.

In addition, four site visits were completed at different co-operatives, during which we informally engaged with members. These provided additional context for understanding how different co-ops could bring change within the economy in England. Finally, documentary sources relevant to the co-ops that participants were members of or talked about were collated and content analysed to provide further contextual insight into their operations (*n* = 19).

Ethical approval was granted by University of York Health Sciences Research Ethics Committee (REF:HSRGC/2024/606/B).

Each interview was recorded, transcribed, and entered into NVivo 14. Data analysis employed Thematic Analysis (Braun and Clarke 2006). The process began with familiarization, involving iterative reading of the transcripts and initial free-coding in NVivo to inform emerging interpretations. Using NVivo also enabled the ease of data retrieval and cross-referencing during the subsequent analytic process. Crucially, issues of health, defined as the ‘capabilities for everyday living’ and power to live a life you value, that were important to participants were identified inductively from the data, based on what participants described about opportunities, resources or experiences that related to their health and wellbeing, including forms of control that enabled or undermined what they did in their everyday.

Analysis progressed through focussed memo-writing and constant comparison, which was integral to our interpretation process to record and share emerging analytic ideas (Gibbs 2007). The dimensions of ‘space’ described above (e.g. political- wider power environment, relational-impetus, temporal-durability) were used as sensitizing concepts to guide this memo-ing, helping to identify and understand how cooperatives were perceived to work. An emergent typology of different co-ops was identified during this process. Separate analytic memos were subsequently developed for each co-op. As analysis progressed, distinct geometries of power became evident, leading to the identification and refinement of three co-op types (see the findings section below).

Insights from documentary sources and site visits were used to question or support emerging findings captured in the memos. Analysis continued through team discussion of each co-op type until an overall coherent story had emerged to describe the findings, and agreement was reached within the team about a set of general propositions relating to the data (Yin 2009). The memos produced are synthesized into the key findings section of this paper.

Pseudonyms are used in the findings section to preserve the anonymity of our research participants and the food co-ops we are writing about. To preserve the anonymity of the co-ops we focused on, we only refer to documentary sources by number, the co-ops’ pseudonym and date (e.g. doc.3, Maple Street Co-op, 2025), not to the original source document.

## Findings

We identified three different types of co-ops: (i) established and scaled worker co-ops, (ii) emanant worker co-ops, and (iii) community organizing co-ops. All involved ‘worker’ members (e.g. people who had specific roles, carried out shifts, took part in decision-making) though not all workers in the three co-ops were paid—some gave their labour voluntarily. Yet, each created conditions for sharing power so that members could exert control in their lives and particularly over their material and political environment (see summary Table 1). The co-ops also created a power geometry that created other opportunities for meaningful connection and purpose, which co-op members valued as important for their wellbeing. As we explain below, however, the contribution that each co-op made towards building these important health-promoting capabilities, and their prospects for contributing to sustainable change within or transformation of the economy in England were constrained by the power dynamics surrounding them.

**Table 1:** Summary of findings.

Co-op type	Power foundations (impetus, origin, relational scale)	Capabilities for health	Challenges for health	Key constraining power dynamics
**1. Established and scale worker co-ops** (e.g. Equity Foods)	Long historical roots (25+ years). Impetus: ethical trading, environmental activism, ‘all in it together’ philosophy Expansive relationally (+50 worker members, national-global food supply chains).	Control—equal income and security: same wage/hour regardless of role; job security for many members—with some exceptions (see challenges) Control—decision-making with purpose: flat structure, collective decision-making and influence (e.g. over pay, pensions, ethical food supply chains, environmental issues) important for wellbeing.	Capitalist contradictions and reproduced inequality: Perceived need to compete at scale leads to use of casualized/temporary (non-owner) labour, excluding some workers from full agency-control/status at work. Carry-in of wider societal inequalities (race, gender) into co-op.	Political/temporal: Entanglement with global capitalism forces compromises original values over time, with implications for worker capabilities for health.
**2. Emanant worker co-ops** (e.g. Gather & Grow)	Nascent/emerging (last 15 years), small scale (less than 20 members), relatively localized links in food economy Impetus: Environmental concerns, desire for value-aligned livelihood, non-hierarchy—links to co-operative culture.	Control—decision-making with purpose: Shared decision-making over tasks and time, and links to mental wellbeing Affiliation and wider welfare: provision of community learning and mental health resilience services (e.g. outdoor food-related programmes) to bring health benefits beyond members.	Financial precarity and reproduced inequality (relational exclusions): Reliance on short-term grants/local state for income, often requires unpaid or supplementary labour—leads to relational exclusions as economic necessity creates a subtle barrier to entry and risks limiting ethnic/socio-economic diversity among workers and who benefits from developing capabilities for health.	Political/temporal/relational: precarious durability due to reliance on relationships with state funding cycles, restricts who can afford to enter and sustain the space and benefit from health promotion potential.
**3. Community organizing co-ops** (e.g. Brickworks)	Relatively informal, and locally anchored, with direct action focus. Impetus: working-class mobilization, control over access to affordable food supplies.	Control—decision-making and direct income savings: making decisions over bulk buying-distributing in food supply chains leaves members with more financial resources and control in everyday life Solidarity and political action: site for strengthening social connection and mobilizing around broader local issues, including those directly relevant to wellbeing (e.g. laundry, childcare).	Market entanglement and reproduced inequality: Use of corporatized surplus food distribution routes to subsidise low costs, operating inside a failing food system. Durability reliant on unequal, unpaid (often gendered) labour, risks reproducing societal inequality.	Political/relational: operates on a small-scale, residual to the state and within a competitive, failing food system.

### 1) Established and scaled worker co-ops

#### Power foundations: scale, longevity and capitalist tension

Borrowing from Powell (2021), ‘established and scaled’ worker co-ops had long historical roots and a level of relational durability or permanence within the economy in England. They were relatively expansive relationally, in terms of the number of worker owners who occupied them (i.e. over 50) and their networks within the food economy (i.e. number of customers, domestic and global supply chains).

Participants spoke to us about one of three specific examples of this type of co-op (Equity Foods, Local Exchange, Maple Street Co-op). Each had a unique history, but the impetus for producing each was more than 25 years ago, driven by people with interests and/or connections to wider environmental, activist or community development movements. Founding members had interests in issues such as ethical trading, ‘taking better care of the environment’ and community (i.e. an ‘all in it together’ philosophy—doc. 2, Local Exchange, 2025). This was reflected in the historical characteristics of each co-op: all were concerned with selling ethical goods (e.g. fairly-traded, sustainable, organic, and/or plant-based), either for bulk wholesale and/or retail, and in supporting their wider communities (doc.1 Equity Foods, 2021; doc.2 Local Exchange, 2025; doc.3 Maple Street Co-op, 2025). These characteristics and values continued to be reflected in the public aspirations of members, as illustrated in Maple Street Co-op’s ethos of ‘ensuring that every decision reflects our values: ecology, social justice, and co-operation’ (doc.3, Maple Street Co-op, 2025).

Yet, each co-op had expanded considerably over the intervening years, with not only many more co-owner worker members but also, at the time of writing, multi-million-pound turnovers within global food supply chains. This scale had introduced a fundamental tension with implications for health, with global connections causing members to ‘very much rub up against capitalism’ (Int. Will, Equity Foods, March 2024) and necessitating a ‘continual balancing act’ to make decisions in ways that respected the control of members and to trade in ways that were ethical and sustainable (doc.13, Maple Street Co-op, 2025).

#### Capabilities for health: equal income and control

The values of each co-op were reflected in the dynamics of who entered the space and with what interests (‘there are a number of us [here] that have been or still are climate activists’, Int. Will, Equity Foods, March 2024) and in how each was reproduced and governed by current members, shaping who could enter and develop ‘capabilities for everyday living’ (cf. WHO 1986). Most particularly, the idea of ‘all being in it together’ was reflected in income equality. In all three examples, all permanent worker members were able to earn the same wage per hour regardless of their role (e.g. whether in the warehouse, driving, or finance). Members were also able to take on different roles to minimize status boundaries at work (e.g. the head of marketing might work a warehouse shift during the week). As Eli noted, this was important for wellbeing:

‘If you just look at it from the amount of very well paid manual labour jobs that [this co-op has] created… in [area], that’s massive… you don’t have people getting paid that kind of money and that kind of job security within those sectors’ (Int. Eli, worked with co-ops, March 2024).

In addition, there were conscious efforts to ensure members were able to exert influence over decisions; whether through formal representative (e.g. Board, surveys) or informal ‘civic’ spaces (e.g. within the canteen, warehouse/shop floor) within the co-op.

A distinguishing feature of this type of co-op was a ‘flat’ formal structure, which meant that each permanent member, at least formally, had the same potential power to exert control over strategy, including, for example, by discussing and reaching a consensus on issues like pay, annual leave, sick pay, pensions, and workplace health (e.g. investing in mental health support, supporting members with childcare). Members were also able to exert influence within food supply chains (e.g. shaping decisions about what products to stock and from who/where—small businesses, fair trade suppliers), in relation to climate change (e.g. ‘climate vetting’ suppliers, investing in a renewable energy supplies); and in local neighbourhoods (e.g. choosing to invest profits or a % of the wage bill in solidarity with local community initiatives or in the Global South). As Will noted, enabling people to have control over work in these ways ‘is a big thing’ (Int. Will, Equity Foods, March 2024) for people’s health and was reflected in people continuing to occupy these spaces: ‘we don't see a lot of people leaving’ (doc.12, Equity Foods, 2020). The co-op's historical commitment to environmental and ethical trading values was important here, enabling members to align their daily work and collective decisions with the capability to connect to nature and concerns for the environment.

#### Challenges for health: casualization and the reproduction of inequality

Despite this health-promoting potential, there were issues and challenges, including psychosocial costs that came with co-ownership and deep involvement in decision-making (‘caring costs’), as well as physical health challenges for members due to the nature of wholesale work. Will spoke, for example, about the physical stresses of lifting and shifting in a warehouse:

‘… it's just not easy, like, working in a warehouse from 2:00pm until 10:00pm or on a night shift picking customer orders, 25 kilo sacks… it's heavy and it's hard’ (Int. Will, Equity Foods, March 2024).

The health-promoting potential of these co-ops was also challenged as they had scaled within a wider capitalist system. The original impetus had ‘creaked and slipped’ (Powell, 2021) over time, leading members to ‘live’ the contradiction during decision-making—for example, in decisions about casualization and ‘giving back’ to communities. In Equity Foods, Will explained that ‘at some point along the way, we started picking up people for… temporary work’ to meet needs in busy periods and ‘partly because members don’t necessarily want to do the less desirable shifts or the less desirable work which is physical’ (Int. Will, Equity Foods, March 2024). At the time of writing, one third of those employed in Equity Foods had temporary, fixed-term (e.g. 12-month) contracts, particularly for less attractive driving and picking roles. Local Exchange also took on casual workers for similar reasons (doc.5, Local Exchange, 2025). While this kind of casualization was framed as being about enabling people to work as and when they wanted to, it was clear that permanent co-owner members had exercised their ‘power over’ others, excluding them from the same status, decision-making rights and income security, and thus from important capabilities to be healthy.

Here then, while status differences in job roles had, to an extent, been ‘designed out’, these co-ops re-created some of the hierarchical patterns of social relations, income insecurity and occupational segregation between ‘owners’ and ‘employees’ found within the wider economy (Harvey 2002) which are known to undermine, rather than promote health. Inevitably, wider unequal societal power dynamics were also carried into these co-ops:

‘there might be race and gender inequalities, there might be experiential inequalities, age inequalities, you know, all these different inequalities get carried in’ (Will, Equity Foods, March 2024).

Similarly, Eli questioned whether decisions made about the way co-ops ‘gave back’ to communities was ‘as creatively distributed’ as possible, noting that while co-ops were generous and gave back a lot:

‘…you can end up cycling co-op money into charity money and yet there’s quite a bit of thinking around why charities are a bit problematic… the way they get tax breaks and the way they get support [are hierarchical]’ (Int. Eli, worked with co-ops, March 2024).

### 2) Emanant worker co-ops

#### Power foundations: nascent agency and non-hierarchy

The second category of co-ops identified were ‘emanant worker co-ops’. We chose the term ‘emanant’ to reflect their nascent and emerging character, which distinguishes them from the more established co-ops discussed above. These co-ops were also smaller and less scaled relationally: fewer workers occupied them (typically less than 20) and their relational networks within the food economy were more localized (e.g. extended only to nearby cities or regions).

The impetus for producing these co-ops was within the last 15 years, with all described by their workers as being ‘in development’. As Dan described it, ‘we are working out how to work together’ (Int. Dan, Gather and Grow Co-op, March 2024). All workers we spoke to were founding members and each indicated that the creation of their co-op was driven by environmental (e.g. climate, biodiversity) concerns and an interest in making a difference for people and the environment. This impetus was reflected in a focus on carrying out food-related activities for a social purpose (e.g. managing land for food growing whilst promoting community learning, using food growing to support social connection or mental wellbeing), as well as providing members with a livelihood that aligned with their values—important capability for health. As Claire explained, she and her co-workers’ focus was ‘being able to have working lives that made sense to us’ and so they could ‘really support one another’ (Int. Claire, Cornerstone Co-op, April 2024). Importantly, central to the emergence of each co-op was links that at least one founding member had, to what Claire called ‘co-operative culture’ (Int. Claire, Cornerstone Co-op, April 2024); for example, through being part of a housing co-op.

#### Capabilities for health: control, affiliation and wider welfare

Central to the design of these, co-ops was a commitment to non-hierarchy and shared decision-making. All worker members earned the same amount and were equally involved in decision-making. Dan and Alicia explained that having direct control over what you did and how you spent time at work was an important capability for them, which positively affected their own mental wellbeing (Ints. Dan, Alicia, Gather and Grow Co-op, March 2024). However, the small relational size of these co-ops introduced challenges in achieving this ideal. As Rich explained, finding time to work on things they wanted could be difficult due to limited resources and strengths to draw upon, including for informing practical decisions. This meant that, at times, although it was an equal space, it could sometimes *feel* hierarchical—given that some people did admin or payroll or day-to-day delivery of services (Int. Rich, Fair Harvest, March 2024).

Given that the impetus for these co-ops was environmental and social good, a notable aspect was their engagement in local welfare provision. They provided food-related services linked to fundamental rights, such as health (e.g. providing wellbeing services) and education, sometimes helping local authorities fulfil their statutory obligations. For example, Gather and Grow Co-op organized food-growing workshops in schools, focused on creating conditions for school children to play, learn about environmental issues and develop attachments to nature. Fair Harvest and Cornerstone Co-op ran outdoor, food-related personal wellbeing and mental health resilience courses, creating conditions for communities who are marginalized (e.g. relating to low income, seeking sanctuary) to build social connection, strengthen emotional development and security, and build collective resilience to disadvantage and anxiety. While these examples represent supporting people to develop important capabilities to be healthy, the involvement of these co-ops in welfare delivery raised questions about whether they were delivering short-term ‘health interventions’ rather than promoting long-term health through structural mechanisms of agency, control, and solidarity.

#### Challenges for health: financial precarity and exclusions

At the same time, the involvement of these co-ops in welfare provision meant that they were quite financially precarious. While some managed to generate their own income (e.g. through running courses or selling trees), workers were reliant on the local state (e.g. the local council’s public health team) or grant funders for their income, profoundly shaping the durability and permanence of the co-op. The precarious funding landscape was a major issue. As Rich noted, ‘the funding landscape is a challenge, and it is relentless having to constantly apply for funding short-term goals’ (Int. Rich, Fair Harvest, March 2024).

Furthermore, several participants questioned whether local authorities or other grant funders understood or valued the co-op model in comparison to more hierarchical charities that are not grounded in solidarity. Dan at Gather and Grow Co-op, for example, felt funders sometimes expected them to do things cheaply or for free by relying on volunteers (as happens in the charitable sector) rather than paying workers a fair wage. For Claire, this precarity meant Cornerstone Co-op was not self-sustaining and often necessitated unpaid labour. Members frequently supplemented their income with other paid employment: ‘we wouldn’t have survived if we didn’t have another income’ (Int. Claire, Cornerstone Co-op, April 2024).

The need for extra income raises critical questions about who can enter and sustain these co-ops, and on what basis, thus limiting accessibility to their health promoting potential for all, particularly those living on a low income in a context of high living costs. Sandy, who supported co-op development, echoed this concern, noting that ‘when you’re talking about people on the ground, with limited resources, trying to develop a co-op, that’s quite a lot of resource and takes a long-time’ (Int. Sandy, worked with co-ops, April 2024). This issue may have been influenced by the relative isolation of these co-ops. While participants spoke about other local co-ops, giving examples of conversations they had had or activities they had done together in the past (e.g. delivering community workshops), they only had limited resources and capacity to deliver services and connect together. There was little sense that these were part of a thriving or connected co-operative ecosystem or political movement.

Resource constraints and financial barriers were reflected in the socio-economic and ethnic diversity of the emanant co-ops. Gather and Grow Co-op and Cornerstone Co-op were predominantly white and occupied by affluent, middle-class members (as self-identified). There was greater ethnic and socio-economic diversity amongst the members of Fair Harvest. Claire, in particular, raised concerns about the workers occupying her co-op’s space: ‘we’re aware that a downside of our organization is that we’ve got four white women working with very ethnically diverse communities’ (Int. Claire, Cornerstone Co-op, April 2024). These issues raise pertinent questions about whether smaller co-ops can be disruptive to the broader political-economic system in England to promote health, or if their potential is limited to building personal, rather than collective capabilities of those privileged enough to be workers in these spaces, or who are able to engage with their services.

### 3) Community organizing co-ops

#### Power foundations: direct action in food systems and local mobilization

The third category of co-ops identified were ‘community-organising’ ones, whose initial focus was bulk-buying and distributing food within local neighbourhoods. These co-ops were less formal than those described above, relatively limited in scale, and whose relational networks were often anchored in a specific urban place (e.g. street, community centre, church, mosque or street in a specific neighbourhood or housing estate). The impetus for producing these co-ops was driven by working class mobilization, including interests in direct action, community benefit and control over access to affordable food supplies.

Participants spoke to us generally about this type of co-op or about specific examples in urban neighbourhoods. One prominent example was Brickworks, which had endured in an inner-city neighbourhood for over 30 years. Described as an ‘experiment in community’, a founding collective of anarchists and squatters had established the co-op as a squatted, non-hierarchical space in a former small shop that they had saved from demolition (Int. Caz; docs.16,17,18 Brickworks, 2024). Other spaces discussed by our participants had emerged more recently (e.g. in the last 5 years) as part of coordinated efforts to politically mobilise working class communities around neighbourhood food buying, given that food is easily ‘relatable’ (Int. Aisha Shared Roots Collective, April 2024): ‘[its] the glue that brings people together’ (doc.14, Shared Roots Collective, 2024).

Reflecting their founding values, these community-organizing co-ops were all quite informally run and self-organizing, with membership quite loosely controlled but requiring everyone ‘to do something’ (Int. Aisha, Shared Roots Collective, April 2024). In Brickworks, food was bought in bulk at wholesale prices and sold ‘as cheaply as we can’ to anyone involved (Int. Caz, Brickworks, March 2024). Reflecting founding members’ anarchist values, specifically the rejection of wage labour and profit, those who worked in the Brickworks shop all volunteered their labour and any mark up on products was only to cover rent and bills, with shoppers also expected to take an active ‘work’ role in retrieving their goods (weighing, bagging, tallying, cleaning up spills) to create a sense of solidarity and mutual connection (Int. Caz. Brickworks, March 2024; doc.18, Brickworks, 2024).

#### Capabilities for health: Solidarity politics and control (income savings and affordable food)

Decisions in these co-ops were typically made by consensus, with members exerting agency and control in deciding what to buy and from whom, and in reflection of their needs and values (e.g. accessing cheap food, buying from other co-ops, taking surplus food or avoiding suppliers with poor working conditions). In this way, the health-promoting potential of these co-ops extended to exerting control in the food system. However, consensus decision-making was not always easy to achieve in practice. Caz indicated that not everyone in Brickworks attended monthly meetings or replied to emails, making it ‘hard to know if you’ve got the consensus’ (Int. Caz, Brickworks, March 2024). Organizing around bulk buying also offered a route for individuals to achieve a more equal income, as members made savings, leaving them with more financial resources to access other things they needed. At the same time, they were sites for strengthening social connection, enabling members to live towards one another and connect across perceived social divides. As Aisha explained, ‘you will have in the same room, the slightly bigoted white old man and the migrant mum and they have a common interest and they actually now speak to each other… and our community is stronger for that’ (Int. Aisha, Shared Roots Collective, April 2024).

These co-ops could also serve as sites for broader health-promoting change, particularly when supported by a wider community development infrastructure (e.g. community workers, connections to other community spaces). While many were, as Aisha put it, ‘just doing their own thing’ (Int. Aisha, Shared Roots Collective, April 2024), we identified examples of members exerting influence over other issues in their lives or local area, including: people supporting each other with childcare, organizing clothes swaps, and establishing a community laundry to mitigate the expense of running a washing machine and address damp homes. In this way, these were spaces that had awakened an ‘alternative’ form of politics: ‘we call it politics through action’ (Int. Aisha, Shared Roots Collective, April 2024).

#### Challenges for health: gendered labour and state-market entanglement

A number of significant challenges threatened the durability and impact of community organizing co-ops. Despite the focus on working-class mobilization, participation was not always accessible to low-income members, with some people’s needs excluded. For Brickworks, Caz noted: ‘I think there definitely are people that have low incomes and they come…. It’s a mixture of people that are local… But I wouldn’t say that, like, it wouldn’t be that easy to afford if you didn’t have an income’ (Int. Caz, Brickworks, March 2024).

Ambitions to exist outside of, or to challenge, the corporatized food system were also often difficult in practice. Community co-ops involved in the Shared Roots Collective, for example, sometimes connected into established corporate-charitable routes for surplus food redistribution. However, this had become a competitive market in some areas, with some ‘on a waiting list to get surplus food’ (Int. Aisha, Shared Roots Collective, April 2024). Whilst this was a route to food access for people on low incomes, it also illustrated how these community co-ops operated inside a broken food system. As Sandy commented, ‘the idea that people are currently surviving on food surplus which is horrific, an awful model… it’s just perverse’ (Int. Sandy, worked with co-ops March 2024).

Finally, the durability of these co-ops was highly reliant on the collective mobilization of communities ‘which is a lot of intense work’ (Int. Sandy, worked with co-ops March 2024). It was noted that this reliance often resulted in an unequal gendered division of unpaid labour—a significant finding that risks reproducing wider societal inequalities. As Aisha commented, this kind of community action, ‘it’s mainly production done by women’ (Int. Aisha, Shared Roots Collective, April 2024; doc.15 Shared Roots Collective, 2024). Furthermore, the long-term survival of even enduring examples like Brickworks had been underpinned by state-backed material support (e.g. free lease, rent subsidies, access to community buildings for meetings). As a result, difficulties in scaling the impact of these co-ops had been compounded by cuts to local Council budgets in recent years due to austerity policies in England and the wider historical decline of co-operative infrastructure, making structural support difficult to secure (Int. Eli, worked with co-ops, March 2024).

## Discussion

Our research offers a novel contribution to literature on health promotion and creating a healthier economy. As far as we are aware, at the time of writing, it is the first paper to explore the role of co-op power dynamics in promoting public health. Our findings show how all three of the co-op types that we identified in the research—established-scaled, emanant worker, and community organizing—create conditions for sharing power, so that members can exert control in their lives. This is a crucial health capability, core to health promotion, but one that is often eroded by conventional market structures and overlooked in public health scholarship (Popay *et al*. 2021). Capability for control is realized through having opportunities to participate in influencing decisions with status and recognition at work, in food supply chains, and in local areas, and through accessing affordable food and a more equal income. Importantly, the co-ops also created opportunities for meaningful connection and purpose, which are also important for wellbeing. Our analysis also revealed, however, that the public health potential of food co-ops has limits, with wider transformative change severely constrained by the wider political, economic and social context in which they operate. Although these findings are limited to one sector of the economy (food) in England, the potential for co-ops to contribute towards creating healthy capabilities, particularly in terms of meaningful control over their environment, can be explored further in future research, and in other sectors of the economy in which co-ops operate (e.g. energy, social care, housing) and other country contexts.

### Operating ‘within’ rather than transforming the current order

While we found that co-ops appear to be ‘spaces of possibility’ for health (Cornwall and Coelho 2006)—as was suggested by Wilkinson and Pickett (2009) in the Spirit Level—their entanglement with the state and dominant economic system, combined with their relative isolation from one another, limits the realization of their health-promoting potential. This finding resonates with wider literature on co-ops, which has shown how co-ops are not ‘cleared’ of the power dynamics and inequality-producing practices that exist within society and the capitalist economy (Cornwall 2002, Powell *et al.* 2021). This unavoidable constraint of operating within the current order limits their transformative capacity (Spicer and Kay 2022). In our findings, we illustrated how forms occupational segregation and exclusion from agency were reproduced in established and scaled worker co-ops, albeit on a limited scale, despite these being conditions that co-ops are often founded to overcome, and which can be fundamental to their identity as organizations (Sobering *et al*. 2014, Sobering 2016). This ‘contradiction’ of ‘being beyond and simultaneously within’ the current order is reflected in other work on co-ops (Homs and Narotzky 2019:133 in Plender 2022) and in other documented compromises, such as how some communities have come to rely on corporatized surplus food redistribution routes to subsidise low costs within an unfair food system (Lambie-Mumford and Kennedy 2025).

Our findings also showed how the impetus of a co-op, and the resulting power dynamics that ensue, can significantly determine who can enter and on what terms, with some limits to accessibility. Here, for example, the small-scale and financial precarity of emanant worker co-ops and reliance on the local state meant that workers frequently required supplementary income, creating a subtle economic barrier to entry and risking exclusions, particularly for communities living on a low income from building capabilities for control over what happens in their lives; despite it being these communities whose health and welfare these co-ops sought to improve. The structural reality of resource scarcity and small scale also led to role differentiation and *feelings* of hierarchy. These are issues that have been raised in other work on co-ops, which have shown how existing forms of power (the ‘old rules of the game’) shape participation, preventing certain people from entering (Sobering 2016, Powell 2021). The reproduction of gendered divisions of labour in community organizing co-ops have also been found in more formal worker co-ops in other country contexts (Sobering 2016). Our work extends these understandings by showing the implications for health and inequality.

Our findings about the precarious durability of emanant worker co-ops, due to the nature of their power relationships with the state, raises questions about the involvement of these co-ops in welfare delivery and whether they are delivering short-term ‘health interventions’ rather than promoting long-term health through structural mechanisms of agency, control, and solidarity. Similarly, the way established and scaled co-ops have ‘creaked and slipped’ through the labour compromises described above (Powell, 2021) also has implications for the agency, control, and income of those involved in these spaces, and thus for health promotion. These issues reflect the power geometries within which co-ops and their workers are located, at a particular moment in time. Previous research has highlighted similar temporal-political challenges for co-ops, including, for example, how changing government attitudes towards them can link co-ops to missions antithetical to their goals (e.g. to The Big Society and austerity agendas in the UK) and with deleterious effects, including their depoliticization (Costa Vieira and Foster 2022).

### Towards a more transformative future?

Overall, our findings show that co-ops have real potential to promote health, but with limits. They are ‘alternative’ spaces within the economy that actively *pre*distribute and ‘design in’ fairer allocations of income, social status, and control for their members (at work, in food systems and local areas), which are important capabilities to be healthy (Ventakapuram 2011, Popay *et al*. 2021). However, the relative isolation of individual food co-ops in England, combined with internal struggles against the constraints of the current economy, limits their ability to achieve systemic disruption and ‘design out’ inequality on a broader scale. They must co-exist with and also resist the very inequality-generating forces they seek to replace.

Without broader collective mobilization to scale out, cross-coordinate and develop a wider, supportive co-operative ecosystem that can help co-op spaces to thrive, it is difficult to see how they can ‘reach a scale that has [transformative] impact’ for the economy or for public health (Allen *et al*. 2003, Hoover and Abell 2016:2; Sutton 2019). This is a fundamental tension inherent to ‘alternative’ institutions globally: the struggle to build transformative spaces within the dominant system (Gritzas and Kavoulakos 2016). A reliance on unpaid, often gendered, labour, difficulty in ensuring inclusion of the most marginalized, and the need to ‘creak and slip’ to survive market pressures, are challenges facing co-ops worldwide (Powell 2021; Spicer and Kay 2022).

Realizing the public health potential of co-ops will require more than localized resilience. It necessitates a global commitment to structural political and financial support to develop diverse cooperative ecosystems, to allow these ‘pockets of possibility’ to scale out principles of democracy, control, and solidarity across the globe and food system. To support such efforts and more fully understand the challenges experienced by food co-ops in particular will require research that not only fully engages people who are involved in them about the challenges they face in particular places but also more fully explores the inter-connections between different types of co-ops that they engage with (i.e. multilevel and participatory action research), to identify practical ways to insulate and grow the kind of food and cooperative ecosystems that could enable health to thrive.

## Data Availability

The data underlying this article cannot be shared publicly to protect the privacy of the people who participated in the study and the food co-ops we are writing about. Anonymized data will be shared on reasonable request to the corresponding author.
